# Comparative proteomics revealed duodenal metabolic function associated with feed efficiency in slow-growing chicken

**DOI:** 10.1016/j.psj.2022.101824

**Published:** 2022-03-08

**Authors:** Pramin Kaewsatuan, Chotima Poompramun, Satoshi Kubota, Jirawat Yongsawatdigul, Wittawat Molee, Pekka Uimari, Amonrat Molee

**Affiliations:** ⁎ School of Animal Technology and Innovation, Institute of Agricultural Technology, Suranaree University of Technology, Nakhon Ratchasima, 30000, Thailand; † School of Food Technology, Institute of Agricultural Technology, Suranaree University of Technology, Nakhon Ratchasima, 30000, Thailand; ‡ Department of Agricultural Sciences, Faculty of Agriculture and Forestry, University of Helsinki, Helsinki, 00790, Finland

**Keywords:** Korat chicken, feed efficiency, slow-growing chicken, label-free proteomics

## Abstract

The Korat chicken (**KR**), developed in Thailand, is a slow-growing breed developed as an alternative breed for Thai chicken producers. The growing interest in slow-growing chicken meat, due to its unique taste, distinct texture, health benefits, and higher broiler welfare have led to higher market demand for KR. However, its low feed efficiency (**FE**) has a significant negative impact on farm profitability. Understanding the molecular mechanism regulating FE allows for designing a suitable selection program and contributing to breeding more efficient chicken for poultry production. Thus, the objective of our study was to investigate the proteome differences and possible pathways associated with FE in male KR using a label-free quantitative proteomic approach. Seventy-five KR males were individually evaluated for FE, and duodenum samples from 6 animals (3 high-FE and 3 low-FE chickens) were collected at 10 wk of age for differential abundant proteins (**DAPs**), protein networks, functional enrichment, and pathway analyses. In this study, we found 40 DAPs significantly associated with FE pathways, including glycolysis/gluconeogenesis, peroxisome, oxidative phosphorylation, tight junction, and cysteine and methionine metabolism. Thus, variations in observed DAPs or genes related to DAPs could be interesting biomarker candidates for selection for higher feed utilization efficiency in chicken.

## INTRODUCTION

Consumers are increasingly interested in meat quality, safety, and animal welfare. These trends favor slow-growing chickens due to the breeds’ meat characteristics, including unique taste, firmer texture, higher nutritional value, and welfare compared to fast-growing commercial breeds (Lusk, 2018). In Thailand, Korat chicken (**KR**), was developed as an alternative meat-type chicken for producers. Korat chicken is a crossbreed between the Thai indigenous Leung Hang Khao chicken and the Suranaree University of Technology (**SUT**) synthetic line.Despite its good meat characteristics, the growth rates and feed efficiency (**FE**) of KR are low, causing low profits (Hang et al., 2018). Thus, to increase its competitiveness in the Thai poultry production market and to offer an efficient alternative breed for small- to moderate-sized Thai farms, improving FE is the most important breeding goal in KR chicken.

Feed efficiency is most often measured as a feed conversion ratio (**FCR**), that is, how many kgs of feed are needed to produce 1 kg of body mass. The heritability of FCR in chickens is moderate, allowing for efficient selection. For example, the estimated heritability for Arkansas broilers was 0.49 (Aggrey et al., 2010) and the estimate for the commercial slow-growing meat-type chicken line was 0.33 (N'Dri et al., 2006). Although improving FCR is possible through selection, it is important to understand the biological basis of FCR given its complexity. Feed efficiency depends on feed intake, energy homeostasis, intestinal structure, and many physiological processes related to the utilization of feed, including intestinal nutrient digestion, absorption, the integrity of the intestinal epithelium, and translocation of intestinal antigens (Richards and Proszkowiec-Weglarz, 2007; Choct, 2009; Nain et al., 2012). Previous studies have shown that high-FE chickens have longer gastrointestinal tracts (Kadhim et al., 2010; Krás et al., 2013; Mabelebele et al., 2014), higher nutrient digestibility (Rougiere et al., 2009; De Verdal et al., 2010), and larger duodenal absorptive villi surface (Nain et al., 2012) than low-FE chicken. The duodenum is a complex organ with an important role in FE, as it regulates the feed digestion process and energy homeostasis (Recoules et al., 2019).

Genome-wide association studies have revealed genomic regions and candidate genes associated with FCR (Mebratie et al., 2019). In addition, transcriptomic studies have revealed pathways related to FE through the digestive function of the duodenum in meat-type chicken (Aggrey et al., 2014; Lee et al., 2015). As the relationship between gene expression levels and their corresponding protein abundance is indirect and the physiological processes are mainly controlled by protein levels (Burgess, 2004), the knowledge from genomic and transcriptomic studies may not be enough to explain the genetic basis of FE. Therefore, proteomic analysis may provide additional insight into the functional mechanisms underlying FE (Kong et al., 2016a; Fu et al., 2017; Fonseca et al., 2019). Currently, little is known about the association between FE traits and proteomics in the small intestinal tissues of chicken, especially in the duodenal part.

A previous study of the pig intestinal proteome revealed important pathways associated with small intestinal structures and movements, including the regulation of actin cytoskeleton, focal adhesion, adherens junction, tight junction, and vascular smooth muscle contraction (Wu et al., 2020). The results suggested that these major physiological processes play a key role in maintaining the integrity of the intestinal epithelium, which is important for digestion and absorption capacity. Therefore, we hypothesized that changes in protein function related to physiological and biological processes in the duodenum may contribute to the FE of chicken.

The objective of our study was to characterize and compare the duodenal proteomic profiles of KR with high- and low-FE using quantitative proteomic technology by high-resolution label-free liquid chromatography-mass spectrometry (**LC-MS**). New information concerning the key molecular pathways regulating FE can be applied in selection programs to improve the efficiency of poultry production.

## MATERIALS AND METHODS

### Ethics Statement

The experiment was conducted at the experimental farm of the Suranaree University of Technology (**SUT**), Thailand. All animal protocols were approved by the Ethics Committee on Animal Use of the Suranaree University of Technology, Nakhon Ratchasima, Thailand (document ID UI-02631-2559).

### Experiment Chickens and Phenotypic Data Collection

The birds used in this study belonged to the KR breed. Each KR generation was formed by crossing Leung Hang Khao males and SUT synthetic line females. To produce the set of birds used in this study, 5th generation KR parental birds with the highest body weight were mated together, and 5th generation KR parental birds with the lowest body weight were mated together. At hatching, the birds were sexed using the vent sexing method, wing-banded, and vaccinated against Marek's disease. Thereafter, they were vaccinated following the recommendation of the Department of Livestock development, Thailand. Seventy-five 1-day-old male KR were individually housed in cages (63 × 125 × 63 cm) covered with rice hulls. All birds were given access to feed and water ad libitum in similar environmental conditions. The same diet was provided to all birds throughout the experiment period using a starter diet (21% protein) for birds 0 to 3 wk of age, a grower diet (19% protein) for birds 4 to 6 wk of age, and a finisher diet (17% protein) for birds 7 to 10 weeks of age. A watering line was supplied across the compartment and attached by nipple drinkers to each cage. Total feed intake and body weight gain from 1 to 10 weeks were measured to calculate FCR:

$$\text{FCR}=\frac{\text{FI}}{\text{BWG}},$$

where FI represents the total feed intake from wk 1 to wk 10 (g) and BWG represents the body weight at wk 10 minus the body weight at wk 1 (g).

At 10 wk of age, the chicken were ranked based on their FCR values. Three chickens with the highest FCR (FCR = 3.33, 3.34, and 3.36) and 3 chickens with the lowest FCR (FCR = 1.83, 1.98, and 1.99) were selected as a low-FE and high-FE groups, respectively, and as a group for the proteomic analysis (3 + 3 biological replicates).

### Duodenal Sample Collection

At the age of 10 wk, all birds were slaughtered with electrical stunning and exsanguination after eight hours of fasting. The intestinal tract was immediately removed, and the whole duodenum was collected and stored in liquid nitrogen at −80°C. During the procedure, dissecting instruments were cleaned with 70% ethanol after each individual bird to prevent cross contamination.

### Protein Extraction

The frozen duodenum samples were freeze-dried, crushed to a fine powder, and lysed in 50 mM ammonium bicarbonate buffer (**AMBIC**) containing 8 M urea (Sigma-Aldrich, St. Louis, MO). The lysed proteins were sonicated on ice and isolated by centrifugation at 20,000 g for 10 min at 4°C. Protein samples were diluted with 50 mM ammonium bicarbonate buffer to a final concentration of 1.5 M urea. Protein concentration was measured using the Pierce BCA Protein Assay kit (Thermo Fisher Scientific, Waltham, MA). Then, 100 µg of proteins from each sample were transferred to a 1.5-mL tube. Finally, the protein was reduced for 20 min at 50°C to 60°C with a final concentration of 5 mM dithiothreitol (**DTT**) and then alkylated for 20 min at room temperature in the darkness with a final concentration of 15 mM iodoacetamide (**IAA**).

### Mass Spectrometry of the Protein Samples

Protein samples were digested with 2 µg trypsin (Promega Corporation, Madison, WI) overnight at 37°C. Mass spectrometry analysis was carried out in a Q Exactive Hybrid Quadrupole-Orbitrap Mass Spectrometer (Thermo Fisher Scientific, Waltham, MA) at the Proteomics Unit core facility, University of Helsinki, Finland. The peptides were separated on a C18 reverse-phase column on an 80-min gradient, and the analysis was carried out using higher-energy collisional dissociation (**HCD**) for mass fragmentation and data-dependent acquisition (**DDA**) mode. One technical replicate of each 6 samples was combined to perform the database search. The raw proteomic data sets in the current study are available on the ProteomeXchange Consortium via the PRIDE (https://www.ebi.ac.uk/pride/) partner repository, with the data set identifier PXD027317 (Reviewer account details: Username: reviewer_pxd027317@ebi.ac.uk; Password: pEjo5kFW).

### Protein Identification Analysis

The raw data from Orbitrap mass spectrometry were imported into MaxQuant software version 1.6.5.0 (Cox and Mann, 2008) for peptide matching to MS/MS spectra. Resulting spectra were identified against the Uniport database of *Gallus gallus* reference proteome (34,925 entries, downloaded from https://www.uniprot.org, January 2019 version). The parameters for the protein identification were trypsin specificity; two missed cleavages and methythio (C) was selected as a fixed modification, and oxidation (M) and acetyl (protein N-term) as a variable modification. The initial precursor (**MS**) mass tolerance was set to 20 ppm in the first search and 6 ppm in the main search. Additionally, fragment (**MS/MS**) mass deviation was set to 20 ppm and both peptide and protein false discovery rates (**FDR**) were set to 1%. The MaxQuant label-free quantification (**LFQ**) algorithm was used for quantification (minimum ratio count = 2).

### Differential Proteomic Analysis

The LFQ intensity values generated by MaxQuant (Cox and Mann, 2008) were used in Perseus software version 1.6.5.0 (Tyanova et al., 2016) for statistical analyses and data visualization. Prior to the analysis, we removed proteins identified by post-translation modification, contaminant proteins, or hits the reverse sequence. Only proteins occurring in 2 out of 3 biological replicates in both experiment groups were kept. Label-free quantification intensity values were transformed to a logarithmic scale with a base of 2. Missing values were imputed from a normal distribution (width: 0.3, down shift: 1.8). Student's t-test was used for comparison between the high- and low-FE groups. Proteins with a *P*-value <0.05 were considered DAPs. Hierarchical clustering was performed with DAPs after Z-score normalization.

The visualization of the differences and similarities of the proteomic profiles and DAPs between the high- and low-FE groups was constructed using a principal component analysis (**PCA**) with the ggplot2 (Wickham, 2009) and ggfortify (Tang et al., 2016) packages in R version 3.5.2 (R Core Team, 2020).

### Bioinformatics Analysis of Differentially Abundant Proteins

Gene ontology (**GO**) enrichment, networks of protein-protein interaction (**PPI**), and Kyoto Encyclopedia of Genes and Genomes (**KEGG**) pathway enrichment analyses were performed using the STRING platform (version 10, http://string-db.org) against the *Gallus gallus* database and considering a medium confidence score of 0.4 for interaction (Szklarczyk et al., 2015). The GO enriched proteins and KEGG pathways were considered enriched with a *P* value <0.05, correcting by FDR with Benjamini-Hochberg method (FDR < 0.05).

## RESULTS AND DISSCUSSION

### Performance and Feed Efficiency Parameters

The performances of two KR chicken groups are illustrated in Table 1. As expected, the difference in FCR between the high-FE (1.93 ± 0.05) and the low-FE (3.34 ± 0.01) groups was highly significant (*P* value < 0.01). Moreover, the body weight gain of the high-FE group was significantly higher than that of the low-FE group (*P* value = 0.01), while the differences in feed intake were not significant (*P* value = 0.080). Thus, the differences in FCR could be mainly explained by the differences in functions related to weight gain.

**Table 1:** Growth performance of the high-FE and low-FE groups from 1 to 10 weeks of age (Mean ± standard error).

Traits	High-FE (n = 3)	Low-FE (n = 3)	*P* value^1^
FI (g)	3173.07 ± 209.25	3807.38 ± 168.30	0.080
BWG (g)	1638.08 ± 84.60	1138.93 ± 49.30	0.012
FCR	1.93 ± 0.05	3.34 ± 0.01	<0.01

Abbreviations: FI: total feed intake from 1 wk to 10 wk; BWG: body weight gain; FCR: feed conversion ratio.

1 Comparison between High-FE and Low-FE groups by a t-test.

### Duodenal Proteome Identification

**Figure 1:**
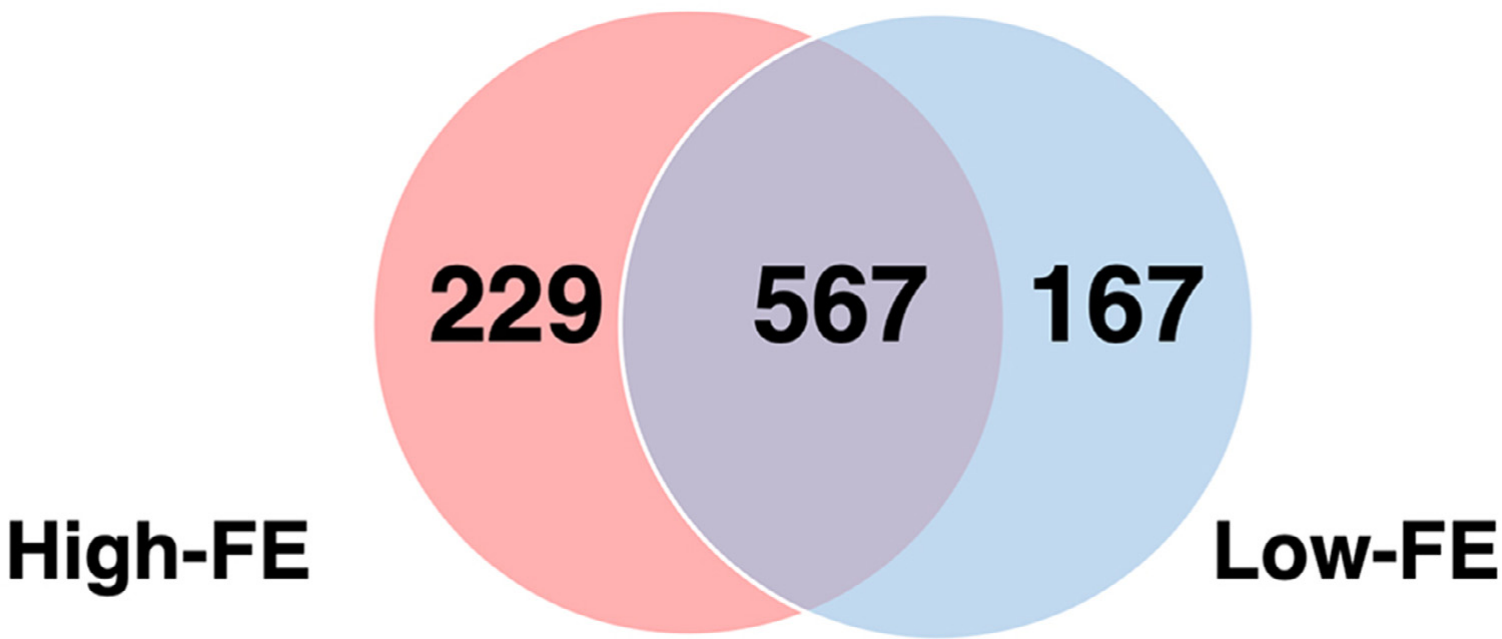
A Venn Diagram representing the number of identified proteins in the duodenal tissue of the Korat chicken that were unique for the high-FE (left) or the low-FE (right) groups, or were common for both groups (center).

A total of 1,013 proteins were initially identified by high-throughput proteomics analysis after eliminating any unnecessary or incorrect protein identifications. Summary information about mass spectrometry analysis can be found in the Supplementary Table 1. Out of the 1,013 identified proteins, 567 proteins were common for both high- and low-FE groups, constituting 56% of the total proteins identified, while 229 (23%) proteins were present only in the high-FE group and 167 (16%) proteins in the low-FE group (Figure 1).

The results suggest that the variation in FE is reflected at the type and level of the duodenal proteome. In contrast to a previous proteomic study, which characterized altered mitochondrial proteins on the skeletal tissue of male broiler exhibiting high-FE and low-FE phenotypes (Kong et al., 2016a), our study is the first to investigate the alteration in the duodenal tissue of a slow-growing chicken using a label-free method, which can provide comprehensive information on FE in chicken.

### Differential Proteomic Analysis Between KR Chicken With High- and Low-FE

Out of the 567 common proteins, 355 proteins were present in 2 out of 3 biological samples in both groups (Supplementary Table 2). A PCA plot was conducted to illustrate the similarities and differences in the proteomic profiles of the high- and low-FE groups. The result revealed that principal component 1 (PC1) explained over 43% of the variance in proteomic abundance. Moreover, the low-FE samples were more closely clustered together, whereas the high-FE samples were scattered, indicating more natural biological variation in protein abundance in the high-FE group than in the low-FE group (Supplementary Figure 1).

**Figure 2:**
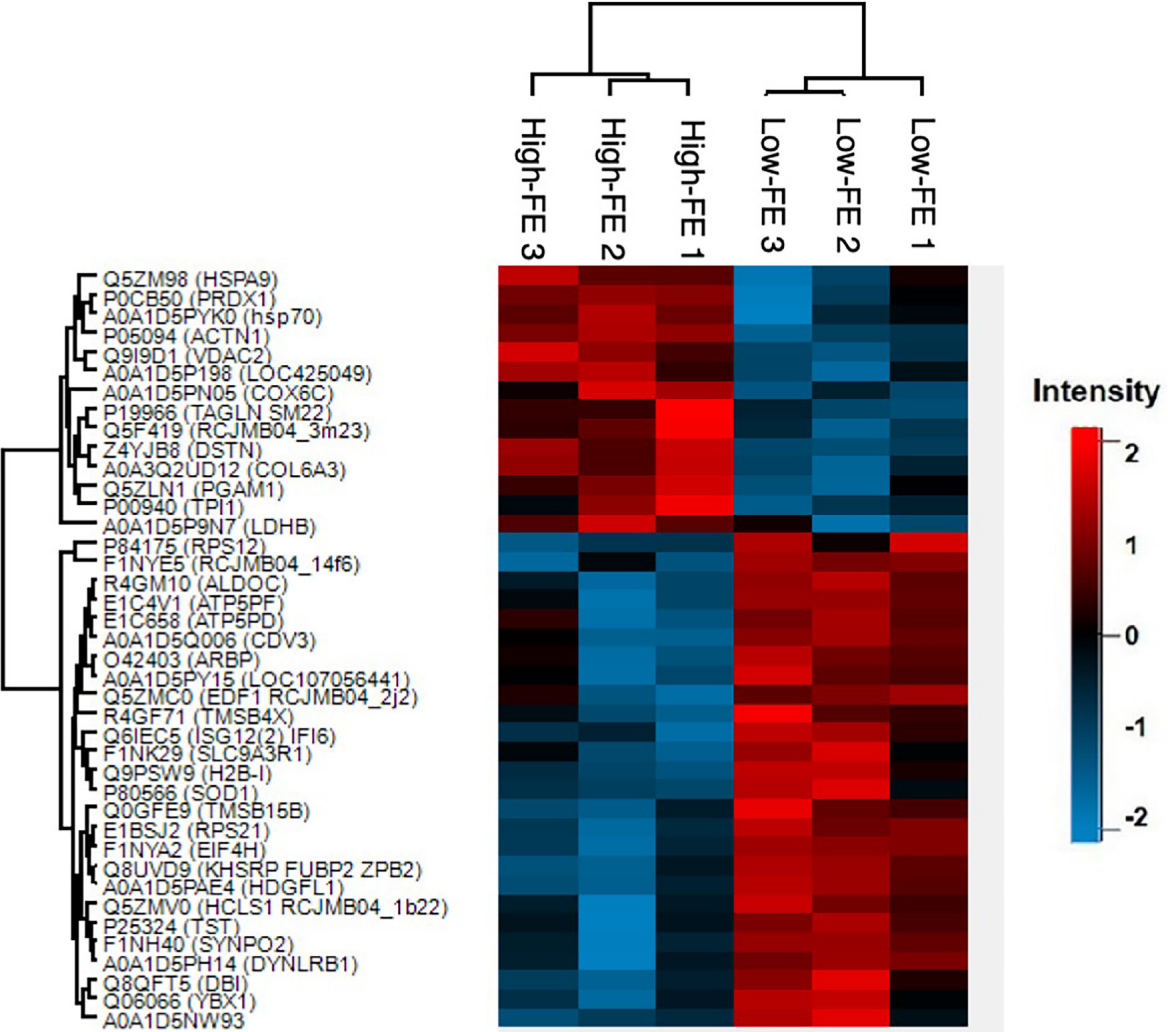
Dendrogram representing the hierarchical clustering of the 40 DAPs in the high-FE and in the low-FE groups. Abbreviation: DAPs, differentially abundant proteins.

The differential protein abundance analysis revealed 40 DAPs that had significantly different abundances between the high- and low-FE groups based on *P* value < 0.05 (Table 2). The hierarchical clustering of the DAPs is illustrated in Figure 2, which showed clear discrimination between the DAP clusters of the 2 FE groups. Within the 40 DAPs, 14 proteins had high abundance in the high-FE group and 26 proteins had high abundance in the low-FE group. Moreover, despite the high level of natural variation in the high-FE group, the PCA plot of the DAPs also showed a clear separation between the two groups, providing evidence that these DAPs are appropriate for group separation (Supplementary Figure 2).

**Table 2:** List of 40 DAPs between the high- and low-FE groups.

Uniprot ID^1^	Protein name	Gene nam	*P* value	FC
Q5ZM98	Stress-70 protein	HSPA9	0.043	−0.55
P0CB50	Peroxiredoxin-1	PRDX1	0.025	−0.69
A0A1D5PYK0	Heat shock cognate 71 kDa protein	HSPA8	0.038	−0.78
P05094	Alpha-actinin-1	ACTN1	0.001	−0.66
Q9I9D1	Voltage-dependent anion channel	VDAC2	0.004	−0.59
A0A1D5P198	Tubulin alpha chain	LOC425049; TUBA3E	0.018	−0.77
A0A1D5PN05	Cytochrome-c oxidase activity	COX6C	0.021	−0.38
P19966	Transgelin	TAGLN	0.035	−0.42
Q5F419	VAMP-associated protein	RCJMB04_3m23; VAPA	0.023	−0.37
Z4YJB8	Destrin	DSTN	0.002	−0.56
A0A3Q2UD12	Collagen alpha-3(VI) chain	COL6A3	0.007	−0.86
Q5ZLN1	Phosphoglycerate mutase 1	PGAM1	0.030	−0.38
P00940	Triosephosphate isomerase 1	TPI1	0.045	−0.55
A0A1D5P9N7	L-lactate dehydrogenase B chain	LDHB	0.043	−0.62
P84175	40S ribosomal protein S12	RPS12	0.017	0.51
F1NYE5	HABP4_PAI-RBP1 domain-containing protein	RCJMB04_14f6	0.011	1.92
R4GM10	Fructose-bisphosphate aldolase C	ALDOC	0.006	0.29
E1C4V1	ATP synthase-coupling factor 6	ATP5PF; ATP5J	0.015	0.68
E1C658	ATP synthase subunit d	ATP5PD; ATP5H	0.048	1.33
A0A1D5Q006	Protein CDV3 homolog	CDV3	0.018	2.26
O42403	Attachment region binding protein	ARBP	0.032	1.08
A0A1D5PY15	Coronin	LOC107056441; CORO1B	0.033	0.61
Q5ZMC0	Endothelial differentiation-related factor 1	EDF1	0.036	0.85
R4GF71	Thymosin beta	TMSB4X	0.033	0.65
Q6IEC5	Putative ISG12(2) protein	ISG12(2) IF16	0.017	1.48
F1NK29	Na(+)/H(+) exchange regulatory cofactor NHE-RF1	SLC9A3R1	0.048	1.00
Q9PSW9	Histone H2B-I	H2B-I	0.012	0.53
P80566	Superoxide dismutase [Cu-Zn]	SOD1	0.032	0.45
Q0GFE9	Thymosin beta	TMSB15B	0.015	0.64
P84175	40S ribosomal protein S12	RPS21	0.004	0.50
F1NYA2	RRM domain-containing protein	EIF4H	0.003	0.78
Q8UVD9	Far upstream element-binding protein 2	KHSRP FUBP2 ZPB2	0.006	0.22
A0A1D5PAE4	Heparin binding growth factor	HDGFL1	0.004	1.17
Q5ZMV0	SH3 domain-containing protein	HCLS1	0.035	1.28
P25324	Thiosulfate sulfurtransferase	TST	0.046	0.72
F1NH40	Synaptopodin 2	SYNPO2	0.017	1.60
A0A1D5PH14	Dynein light chain roadblock	DYNLRB1	0.025	0.90
Q8QFT5	Diazepam binding inhibitor	DBI	0.020	0.64
Q06066	Y-box-binding protein 1	YBX1	0.040	0.77
A0A1D5NW93	ATP synthase F1 subunit delta	ATP5PD;ATP5D	0.039	0.43

1 Protein accession number from the Uniprot database (www.uniprot.org). Abbreviations: DAPs: differentially abundant proteins; FC: fold change.

### Functional Enrichment of the DAPs

We conducted functional enrichment analysis to associate the DAPs with their biological processes, molecular functions, and cellular components. The results of the enrichment analysis are presented in Table 3. Among the biological processes, the largest number of DAPs (15 out of 40) were related to cellular process (GO:0009987). The other common biological processes were cellular metabolic process (GO:0044237) (10 out of 40 DAPs) and the regulation of cellular process (GO:0050794) (8 out of 40 DAPs). The 3 most common molecular functions were binding (GO:0005488) (13 out of 40 DAPs), protein binding (GO:0005515) (8 out of 40 DAPs), and catalytic activity (GO:0003824) (7 out of 40 DAPs). The largest number of DAPs in the cellular component were related to intracellular part (GO:0044424) (19 out of 40 DAPs), cytoplasm (GO:0005737) (17 out of 40 DAPs), and intracellular organelle (GO:0043229) (12 out of 40 DAPs).

**Table 3:** Significant Gene ontology (GO) terms of DAPs listed in Table 1.

GO ID	Description	adj. *P* value^1^		Proteins
Biological process				
GO:0006090	Pyruvate metabolic process	3.00E−05		ALDOC, LDHB, PGAM1, TPI1
GO:0051186	Cofactor metabolic process	3.00E−05		ALDOC, HSPA9, PGAM1, PRDX1, TPI1
GO:0009987	Cellular process	0.0001		ACTN1, ALDOC, DSTN, EDF1, H2B-I, HSPA9, LDHB, PGAM1, PRDX1, RPS12, SLC9A3R1, SOD1, TAGLN, TPI1, YBX1
GO:0005975	Carbohydrate metabolic process	0.0003		ALDOC, LDHB, PGAM1, TPI1
GO:0006006	Glucose metabolic process	0.0003		ALDOC, PGAM1, TPI1
GO:0006094	Gluconeogenesis	0.0003		ALDOC, PGAM1, TPI1
GO:0006096	Glycolytic process	0.0003		ALDOC, PGAM1, TPI1
GO:0006754	ATP biosynthetic process	0.0003		ALDOC, PGAM1, TPI1
GO:0006757	ATP generation from ADP	0.0003		ALDOC, PGAM1, TPI1
GO:0009166	Nucleotide catabolic process	0.0003		ALDOC, PGAM1, TPI1
GO:0017144	Drug metabolic process	0.0003		ALDOC, PGAM1, PRDX1, TPI1
GO:0019359	Nicotinamide nucleotide biosynthetic process	0.0003		ALDOC, PGAM1, TPI1
GO:0042866	Pyruvate biosynthetic process	0.0003		ALDOC, PGAM1, TPI1
GO:0046496	Nicotinamide nucleotide metabolic process	0.0003		ALDOC, PGAM1, TPI1
GO:0009168	Purine ribonucleoside monophosphate biosynthetic process	0.0003		ALDOC, PGAM1, TPI1
GO:0019430	Removal of superoxide radicals	0.0003		PRDX1, SOD1
GO:0044237	Cellular metabolic process	0.0004		ALDOC, EDF1, HSPA9, LDHB, PGAM1, PRDX1, RPS12, SOD1, TPI1, YBX1
GO:0009167	Purine ribonucleoside monophosphate metabolic process	0.0008		ALDOC, PGAM1, TPI1
GO:0044248	Cellular catabolic process	0.0012		ALDOC, PGAM1, PRDX1, TPI1
GO:0044271	Cellular nitrogen compound biosynthetic process	0.0019		ALDOC, EDF1, PGAM1, RPS12, TPI1, YBX1
GO:0034101	Erythrocyte homeostasis	0.0021		HSPA9, PRDX1
GO:1901566	Organonitrogen compound biosynthetic process	0.0031		ALDOC, PGAM1, RPS12, TPI1
GO:0034654	Nucleobase-containing compound biosynthetic process	0.0052		ALDOC, EDF1, PGAM1, TPI1, YBX1
GO:1901576	organic substance biosynthetic process	0.0059		ALDOC, EDF1, PGAM1, RPS12, TPI1, YBX1
GO:0055114	Oxidation-reduction process	0.0066		LDHB, PRDX1, SOD1
GO:0050794	Regulation of cellular process	0.0077		COL6A3, DSTN, EDF1, HSPA9, PGAM1, PRDX1, SLC9A3R1, YBX1
GO:0007015	Actin filament organization	0.0088		ACTN1, DSTN
GO:0051171	Regulation of nitrogen compound metabolic process	0.0097		COL6A3, EDF1, PGAM1, PRDX1, SLC9A3R1, YBX1
GO:0080090	Regulation of primary metabolic process	0.0101		COL6A3, EDF1, PGAM1, PRDX1, SLC9A3R1, YBX1
GO:0031323	Regulation of cellular metabolic process	0.0109		COL6A3, EDF1, PGAM1, PRDX1, SLC9A3R1, YBX1
GO:0060255	Regulation of macromolecule metabolic process	0.0109		COL6A3, EDF1, PGAM1, PRDX1, SLC9A3R1, YBX1
GO:0044238	Primary metabolic process	0.0186		ALDOC, EDF1, LDHB, PGAM1, RPS12, TPI1, YBX1
GO:0019220	Regulation of phosphate metabolic process	0.0211		PGAM1, PRDX1, SLC9A3R1
GO:0071704	Organic substance metabolic process	0.0223		ALDOC, EDF1, LDHB, PGAM1, RPS12, TPI1, YBX1
GO:0006139	Nucleobase-containing compound metabolic process	0.0260		ALDOC, EDF1, PGAM1, TPI1, YBX1
GO:0022607	Cellular component assembly	0.0497		ACTN1, H2B-I, HSPA9
Molecular function				
GO:0005488	Binding	0.0004		ACTN1, DBI, DSTN, EDF1, H2B-I, HSPA8, HSPA9, PRDX1, SLC9A3R1, SOD1, TAGLN, TST, YBX1
GO:0005515	Protein binding	0.0024		ACTN1, DSTN, H2B-I, HSPA8, HSPA9, PRDX1, SLC9A3R1, TAGLN
GO:0051015	Actin filament binding	0.0024		ACTN1, DSTN, TAGLN
GO:0016209	Antioxidant activity	0.0049		PRDX1, SOD1
GO:0051219	Phosphoprotein binding	0.0049		ACTN1, HSPA8
GO:0003824	Catalytic activity	0.0066		ALDOC, LDHB, PGAM1, PRDX1, SOD1, TPI1, TST
GO:0016853	Isomerase activity	0.0073		PGAM1, TPI1
GO:0016491	Oxidoreductase activity	0.0117		LDHB, PRDX1, SOD1
GO:0097159	Organic cyclic compound binding	0.0117		DBI, EDF1, H2B-I, HSPA8, HSPA9, TST, YBX1
GO:1901363	Heterocyclic compound binding	0.0117		DBI, EDF1, H2B-I, HSPA8, HSPA9, TST, YBX1
Cellular component				
GO:0005737	Cytoplasm	7.10E-09		ACTN1, ALDOC, CDV3, DBI, EDF1, HSPA8, HSPA9, LDHB, PGAM1, PRDX1, RPS12, SLC9A3R1, SOD1, TAGLN, TPI1, TST, YBX1
GO:0044424	Intracellular part	7.10E-09		ACTN1, ALDOC, CDV3, DBI, DSTN, EDF1, H2B-, HSPA8, HSPA9, LDHB, PGAM1, PRDX1, RPS12, SLC9A3R1, SOD1, TAGLN, TPI1, TST, YBX1
GO:0044444	Cytoplasmic part	0.0001		ACTN1, ALDOC, DBI, EDF1, HSPA9, LDHB, PGAM1, RPS12, TPI1, TST
GO:0043229	Intracellular organelle	0.0004		ACTN1, DBI, DSTN, EDF1, H2B-I, HSPA8, HSPA9, PRDX1, RPS12, SOD1, TST, YBX1
GO:0005829	Cytosol	0.0004		ALDOC, EDF1, LDHB, PGAM1, RPS12, TPI1
GO:0043231	Intracellular membrane-bounded organelle	0.0077		DBI, EDF1, H2B-I, HSPA8, HSPA9, PRDX1, SOD1, TST, YBX1
GO:0001726	Ruffle	0.0098		ACTN1, SLC9A3R1
GO:0043209	Myelin sheath	0.0098		PGAM1, PRDX1
GO:1990904	Ribonucleoprotein complex	0.0106		HSPA8, RPS12, YBX1
GO:0032991	Protein-containing complex	0.0139		COL6A3, EDF1, H2B-I, HSPA8, RPS12, YBX1
GO:0043232	Intracellular non–membrane-bounded organelle	0.0244		ACTN1, DSTN, EDF1, H2B-I, RPS12

1 FDR-adjusted *P*-values. Abbreviations: DAPs: differentially abundant proteins.

The results indicate that most of the DAPs found between high- and low-FE groups relate to many essential metabolic processes that function in the duodenum. Given that the duodenum is the main organ in the nutrient digestion process, the results also reveal the biochemical and physiological aspects of molecular metabolism regulating FE. In addition, our results support previous findings that several physiological processes, for example, feed intake, feed digestion, metabolism, physical activity, and thermoregulation relate to FE (Herd and Arthur, 2009).

### Protein Interaction Network and Enrichment Pathways of DAPs

**Figure 3:**
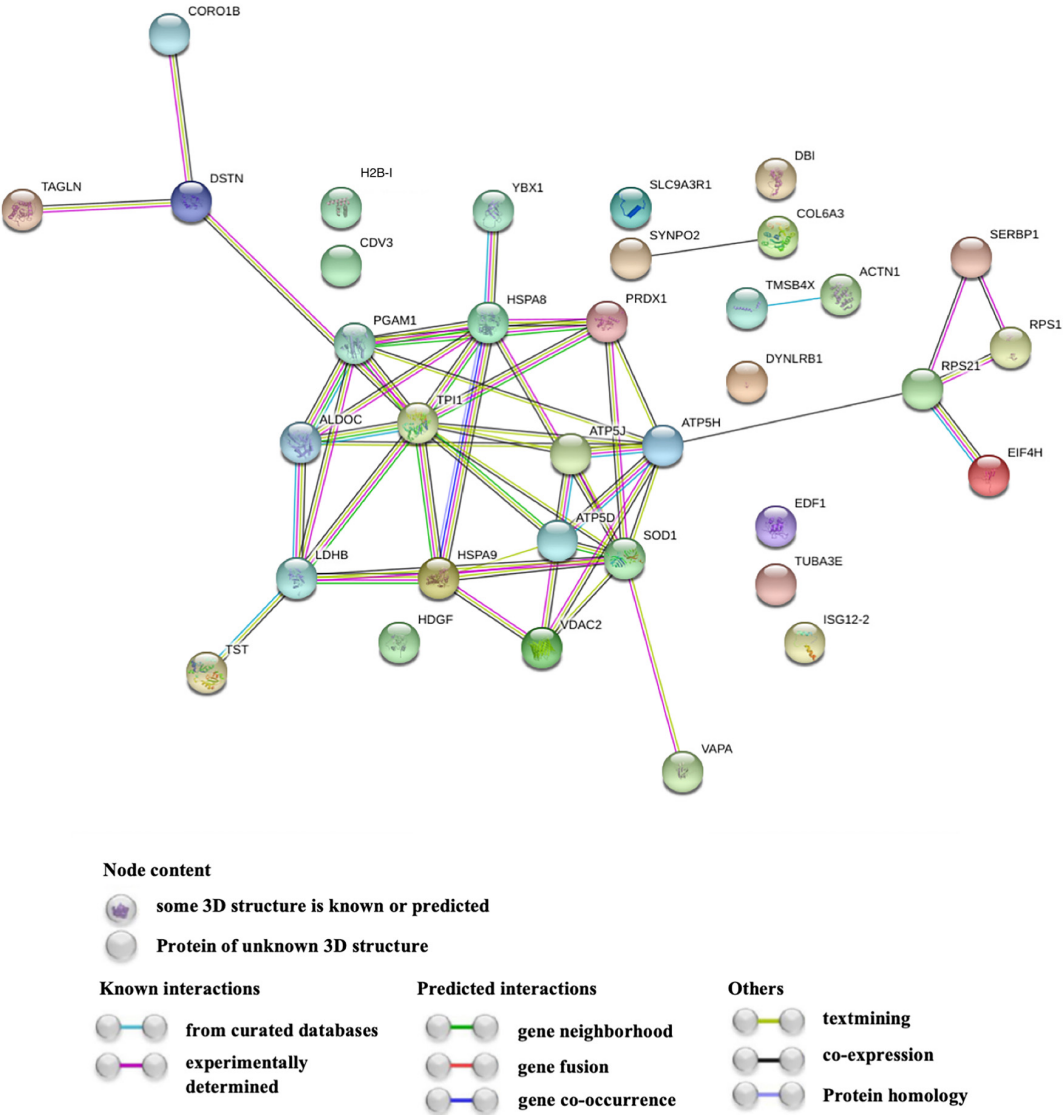
Protein-protein interaction network of DAPs. Nodes represent the DAPs identified with the coding gene symbol, colored nodes indicate the query proteins, and lines represent the connections between the proteins. Abbreviation: DAPs, differentially abundant proteins.

Analysis of the PPI network revealed nine proteins (H2B-I, ISG12-2, EDF1, DBI, DYNLRB1, SLC9A3R1, HDGF, TUBA3E, CDV3) that had no interaction with other DAPs (Figure 3). This may indicate that these proteins may not be biologically relevant or may have independent functions. However, the majority of DAPs interacted with each other and formed clusters, comprising with metabolic enzymes (PGAM1, TPI1, ALDOC, LDHB), cytoskeleton proteins (DSTN, CORO1B, TAGLN), ribosomal proteins (SERBP1, RPS21, RPS1), translational initiation factor (EIF-4H), stress-responsive proteins (PRDX1, SOD1, HSPA8, HSPA9), and electron transport chain proteins (VDAC2, ATP5J, ATP5D, ATP5H), which interacted closely with VAPA, TST, and YBX1. Further, the small components of interacting proteins were also presented in this network, including ACTN1, TMSB4X, SYNPO2, and COL6A3. Such interactions may indicate that these proteins function cooperatively in FE regulation.

DAPs were further explored based on the KEGG pathway to elucidate the metabolic pathway in which these proteins were involved. Five enriched pathways identified are presented in Table 4: glycolysis/gluconeogenesis, peroxisome, oxidative phosphorylation, tight junction, and cysteine and methionine metabolism. Below we have discussed the potential roles of these proteins in functional pathways that could affect FE.

**Table 4:** Enriched metabolic pathways of DAPs.

KEGG ID	Description	Adj. *P* value^1^	Proteins
00010	Glycolysis/Gluconeogenesis	0.0002	ALDOC, **LDHB, PGAM1, TPI1**
04146	Peroxisome	0.048	**PRDX1**, SOD1
00190	Oxidative phosphorylation	0.018	ATP5J, ATP5H, ATP5D
04530	Tight junction	0.018	**ACTN1**, SLC9A3R1, **TUBA3E**
00270	Cysteine and methionine metabolism	0.018	**LDHB**, TST

1 FDR-adjusted *P*-values. High abundant proteins in the high-FE group are marked in bold face. Abbreviations: DAPs: differentially abundant proteins.

#### Proteins Related to Carbohydrate Metabolism

The glycolysis/gluconeogenesis pathway is the most enriched term of DAPs. It is well known that glucose catabolism of the intestinal tract is essential for providing energy during the digestion and absorption process (Fändriks, 2017). In glycolysis, glucose breakdown generates 2 molecules of pyruvate, which is the main glycolytic product that can be oxidized to produce ATP for cellular metabolism through the tricarboxylic acid (**TCA**) cycle and oxidative phosphorylation (Fonseca et al., 2019). Therefore, it was interesting that 3 proteins were among the most abundant in the duodenum of the high-FE group, including triphosphate isomerase (**TPI1**) and phosphoglycerate mutase 1 (**PGAM1**), which are glycolytic enzymes that catabolize glucose into pyruvate (Alberts et al., 2002), and lactate dehydrogenase (**LDHB**), an enzyme that converts lactate to pyruvate (Zhao et al., 2020). These three enzymes are important in the rumen epithelium of inefficient steers (Kong et al., 2016b) and also in the jejunum of low-FCR chicken (Shah et al., 2019). Thus, higher levels of these 3 enzymes in the high-FE group can have a major impact on the pyruvate generation rate, resulting in higher ATP production in the high-FE group compared to the low-FE group. Our result also indicates that high-FE chicken can use more glucose as an oxidative substrate for energy generation in the duodenal epithelium than low-FE chicken.

#### Proteins Related to Cysteine and Methionine Metabolism

Glucogenic amino acids, cysteine, and methionine are the major precursors that can be converted into glucose (Brosnan, 2003). In this study, thiosulfate sulfurtransferase (**TST**) was a DAP in the low-FE group. Thiosulfate sulfurtransferase is an enzyme involved in sulfide catabolism to sulfite, sulfate, and thiosulfate, which is important for producing cysteine from methionine via the transsulfuration pathway (Kohl et al., 2019). Thus, increased levels of this enzyme may provide more compensatory precursors for gluconeogenesis through amino acid metabolism in the low-FE group to meet their energy demands. In our findings, the abundance of aldolase C (**ALDOC**) was higher in the low-FE group than in the high-FE, supporting this assumption. ALDOC catalyzes the reversible dihydroxyacetone phosphate (**DHAP**) and glyceraldehyde 3-phosphate (**G3P**) to form fructose-1,6-bisphosphate (**F-1,6-BP**) in the gluconeogenic direction. Our results are consistent with Zhang et al. (2019), who reported that proteins involved in gluconeogenesis and amino acid metabolism in the livers of low-FE heifers were upregulated. Although previous studies have shown that the carbon transaction process between amino acid and carbohydrate metabolism mainly occurs in the liver and muscles of chicken (Abasht et al., 2019; Yang et al., 2020), our study revealed that high-abundant protein related to the metabolic fate of amino acids and glucose can occur in the intestinal tract of slow-growing chicken. This process may indicate that low-FE chicken tend to adapt to maintaining their blood glucose levels and simultaneously preserving their energetic status for metabolic purposes.

#### Proteins Related to Mitochondrial Oxidative Metabolism and Oxidative Stress

The majority of the energy production of a eukaryotic cell is generated through oxidative phosphorylation in the mitochondrial inner membrane (Bottje et al., 2006). The final phase of oxidative phosphorylation is carried out by ATP synthase or Complex V, one of the components of the electron transport chain. Interestingly, we observed three highly abundant proteins in the low-FE group: ATP synthase-coupling factor 6 (**ATP5J**), ATP synthase subunit d (**ATP5H**), and ATP synthase F1 subunit delta (**ATP5D**). These 3 proteins play an important role in the proton channel of ATP synthase facilitating electron flow through the respiratory chain and providing energy for ATP synthesis. The same proteins were previously reported to be abundant in the rumen epithelium of L-RFI steer (Kong et al., 2016b) and in the breast muscle of L-RFI chickens (Yang et al., 2020). Thus, increased levels of these proteins suggest that the low-FE group may have increased energy production in the form of ATP in its duodenum, requiring a large quantity of energy. In addition, mitochondria are well recognized as a major souce of reactive oxygen species (**ROS**), such as superoxide (**O_2_^•−^**), produced by the electron transport chain during the process of oxidative phosphorylation. Some DAPs in our study are related to cell defense against ROS. For example, superoxide dismutase 1 (**SOD1**), which converts O_2_^•−^ into hydrogen peroxide **(H_2_O_2_**) (Schäff et al., 2012), was more abundant in the low-FE group compared to the high-FE group. The over-production of SOD1 may result in elevated generation of H_2_O_2_, as observed in mitochondrial duodenal tissue (Ojano-Dirain et al., 2004) and in the breast (Bottje et al., 2006) of low-FE broilers. Furthermore, given that peroxiredoxin-1 (**PRDX1**) is important in cellular oxidative stress defense (Jeong et al., 2018), our findings (a lower level of PRDX1 in the low-FE group compared to the high-FE group) suggest that low-FE chickens are unable to remove excessive ROS as effectively as high-FE chickens and thus more likely suffer from oxidative damage than high-FE chickens. Peroxisomal metabolism, which is closely related to mitochondrial metabolism and immune response activation, is reportedly important for improving FE in poultry (Di Cara et al., 2019; Xiao et al., 2021). We thus hypothesized that proteins associated with the peroxisome pathway are responsible for modulating redox imbalance between ROS production and elimination caused by mitochondrial dysfunction, which contributes to oxidative stress in the chicken duodenum. Also, both mitochondrial inefficiency and oxidative stress may contribute to FE variation (Bottje and Carstens, 2009). In agreement with this, duodenal genes related to ROS production were over-represented in inefficient chickens (Yi et al., 2015) and beef cattle (Yang et al., 2021). Moreover, the failure of ROS detoxification can lead to intestinal inflammation and poor absorption (Mishra and Jha, 2019). Therefore, we suggest that greater susceptibility to oxidative stress may be responsible for the poorer FE in the low-FE group compared to the high-FE group.

#### Proteins Related to Intestinal Nutrient Permeability

The tight junction pathway is related to the physiological function of epithelial cells affecting the absorption of nutrients in the small intestine (Choct, 2009). The stability of tight junctions has an impact on the capacity of intestinal barrier permeability. Previous research showed that tight junction integrity and paracellular permeability were associated with the regulation of actin cytoskeleton and intercellular adhesion strength (Bruewer et al., 2004). In our study, three DAPs (ACTN1, SLC9A3R1, TUBA3E) were related to the tight junction pathway. Alpha actinins (**ACTN1**) and the tubulin alpha-3E chain (**TUBA3E**) were highly abundant in the high-FE group. This agrees with a transcriptome study showing upregulation of the genes encoding these proteins in the L-RFI epithelium of beef cattle (Kong et al., 2016b). The overproduction of these proteins suggest that high-FE chickens have greater intestinal integrity and epithelial function than low-FE chickens, possibly also resulting in greater paracellular nutrient permeability.

Tight junctions not only play a key role in nutrient absorption but also form a physical barrier against the external environments of the intestinal epithelial cell to prevent the entry of unwanted organisms, antigens, and toxins (Groschwitz and Hogan, 2009). Damage to the intestinal epithelial barrier can lead to inflammation (De Meyer et al., 2019). Thus, it is important for efficient production and optimal health (De Oliveira et al., 2018). Hypothetically, a better adaptive immune response requires less energy that can be used for growth (Horodyska et al., 2018).

Solute carrier family 9, subfamily A (**SLC9A3R1**) was highly abundant in the low-FE group. SLC9A3R1 is involved in several signaling pathways, such as cAMP-mediated phosphorylation that induces phosphorylation of claudin, a tight junction protein that is the major determinant of the barrier function (Chiba et al., 2008). Related to this, cAMP elevates the barrier function via PKA-dependent and -independent pathways enhancing the junctional immunoreactivity of claudin and changing the barrier function of tight junction proteins (Chiba et al., 2008). Therefore, it is possible that the overproduction of the SLC9A3R1 protein may be related to the physiological adaptation of tight junctions to prevent the failure of its intestinal epithelium barrier function when gut health is compromised.

Based on our proteomics result, changes in metabolic activity, energy homeostasis, oxidative stress, and tight juction appear to play important roles in regulating FE. Previous studies showed that animals with poor FE require more energy for maintaining tissue homeostasis and have less usable energy for growth (Fonseca et al., 2019; De Lima et al., 2020). This may explain why the low-FE group gained less weight when consuming the same amount of feed as the high-FE group. Selecting for better FCR therefore promotes better feed conversion efficiency, growth, and production. In our study, we did not identify pathways involved in nutrient absorption. Dokladny et al. (2016) reported that microvilli is a crucial factor affecting the nutrient absorption capacity of the small intestine. Although our study detected some proteins related to the microvilli, such as plastin-1 (**PLS1**), vinculin (**VCL**), F-actin-capping protein subunit beta (**CAPZB**), F-actin-capping protein subunit alpha (**CAPZA2**), and actin-related protein 3 (**ACTR3**), the abundances of these proteins were either relatively low or did not differ between the two groups. Further studies focused on microvilli are required to profoundly understand the mechanism related to nutrient absorption.

## CONCLUSION

Our results indicate that the different FE potential of slow-growing chicken is related to duodenal metabolism through proteins enriched in five main metabolic pathways: glycolysis/gluconeogenesis, peroxisome, oxidative phosphorylation, tight junction, and cysteine and methionine metabolism. These findings suggest that high-FE chickens have better glucose breakdown to extract energy for cellular metabolism from glycolysis and better tight junction strength of their intestinal epithelium than low-FE chickens. On the other hand, low-FE chickens may need to activate their amino acid metabolism and oxidative phosphorylation to provide more compensatory precursors for gluconeogenesis, to prevent disruption in their intestinal barrier function. These findings provide potential dietary energy-related biomarkers for selection to improve FE in chicken. However, given the relatively small number of biological replicates used in this study, further work is needed to confirm these findings.

## Appendix Supplementary materials

Image, application 1

**Supplementary Figure 1.** Principal component analysis (PCA) plot using 355 proteins from the low-FE (blue) and high-FE (red) groups. Image, image 2

**Supplementary Figure 2.** Principal component analysis (PCA) plot using 40 DAPs from the low-FE (blue) and high-FE (red) groups. Image, image 3

## Footnotes

Supplementary material associated with this article can be found in the online version at https://doi.org/10.1016/j.psj.2022.101824.

## References

[bib0001] Abasht B., Zhou N., Lee W.R., Zhuo Z., Peripolli E. (2019). The metabolic characteristics of susceptibility to wooden breast disease in chickens with high feed efficiency. Poult. Sci..

[bib0002] Aggrey S.E., Karnuah A.B., Sebastian B., Anthony N.B. (2010). Genetic properties of feed efficiency parameters in meat-type chickens. Genet. Sel. Evol..

[bib0003] Aggrey S., Lee J., Karnuah A., Rekaya R. (2014). Transcriptomic analysis of genes in the nitrogen recycling pathway of meat-type chickens divergently selected for feed efficiency. Anim. Genet..

[bib0004] Alberts B., Johnson A., Lewis J., Raff M., Roberts K., Walter P. (2002).

[bib0005] Bottje W., Carstens G. (2009). Association of mitochondrial function and feed efficiency in poultry and livestock species. J. Anim. Sci..

[bib0006] Bottje W., Pumford N., Ojano-Dirain C., Iqbal M., Lassiter K. (2006). Feed efficiency and mitochondrial function. Poult. Sci..

[bib0007] Brosnan J.T. (2003). Interorgan amino acid transport and its regulation. J. Nutr..

[bib0008] Bruewer M., Hopkins A.M., Hobert M.E., Nusrat A., Madara J.L. (2004). RhoA, Rac1, and Cdc42 exert distinct effects on epithelial barrier via selective structural and biochemical modulation of junctional proteins and F-actin. Am. J.Physiol. Cell. Physiol..

[bib0009] Burgess S. (2004). Proteomics in the chicken: tools for understanding immune responses to avian diseases. Poult. Sci..

[bib0010] Chiba H., Osanai M., Murata M., Kojima T., Sawada N. (2008). Transmembrane proteins of tight junctions. Biochim. Biophys. Acta..

[bib0011] Choct M. (2009). Managing gut health through nutrition. Br. Poult. Sci..

[bib0012] Cox J., Mann M. (2008). MaxQuant enables high peptide identification rates, individualized ppb-range mass accuracies and proteome-wide protein quantification. Nat. Biotechnol..

[bib59] De Lima A.O., Koltes J.E., Diniz W.J.S, De Oliveira P.S.N., Cesar A.S.M., Tizioto P.C., Afonso J., De Souza M.M., Petrini J., Rocha M.I.P., Cardoso T.F., Neto A.Z., Coutinho L.L., Mourão G.B., Regitano L.C.A. (2020). Potential biomarkers for feed efficiency-related traits in nelore cattle identified by co-expression network and integrative genomics analyses. Front. Genet..

[bib0014] De Meyer F., Eeckhaut V., Ducatelle R., Dhaenens M., Daled S., Dedeurwaerder A., De Gussem M., Haesebrouck F., Deforce D., Van Immerseel F. (2019). Host intestinal biomarker identification in a gut leakage model in broilers. Vet. Res..

[bib0015] De Oliveira P.S.N., Coutinho L.L., Tizioto P.C., Cesar A.S.M., de Oliveira G.B., Diniz W.J.d.S., De Lima A.O., Reecy J.M., Mourão G.B., Zerlotini A., Regitano L.C.A. (2018). An integrative transcriptome analysis indicates regulatory mRNA-miRNA networks for residual feed intake in Nelore cattle. Sci. Rep..

[bib0016] De Verdal H., Mignon-Grasteau S., Jeulin C., Bihan-Duval E.Le, Leconte M., Mallet S., Martin C., Narcy A. (2010). Digestive tract measurements and histological adaptation in broiler lines divergently selected for digestive efficiency. Poult. Sci..

[bib0017] Di Cara F., Andreoletti P., Trompier D., Vejux A., Bülow M.H., Sellin J., Lizard G., Cherkaoui-Malki M., Savary S. (2019). Peroxisomes in Immune Response and Inflammation. Int. J. Mol. Sci..

[bib0018] Dokladny K., Zuhl M.N., Moseley P.L. (2016). Intestinal epithelial barrier function and tight junction proteins with heat and exercise. J. Appl. Physiol..

[bib0019] Fändriks L. (2017). Roles of the gut in the metabolic syndrome: an overview. J. Intern. Med..

[bib0020] Fonseca L.D., Eler J.P., Pereira M.A., Rosa A.F., Alexandre P.A., Moncau C.T., Salvato F., Rosa-Fernandes L., Palmisano G., Ferraz J.B. (2019). Liver proteomics unravel the metabolic pathways related to feed efficiency in beef cattle. Sci. Rep..

[bib0021] Fu L., Xu Y., Hou Y., Qi X., Zhou L., Liu H., Luan Y., Jing L., Miao Y., Zhao S. (2017). Proteomic analysis indicates that mitochondrial energy metabolism in skeletal muscle tissue is negatively correlated with feed efficiency in pigs. Sci. Rep..

[bib0022] Groschwitz K.R., Hogan S.P. (2009). Intestinal barrier function: molecular regulation and disease pathogenesis. J. Allergy. Clin. Immunol..

[bib0023] Hang T., Molee W., Khempaka S., Paraksa N. (2018). Supplementation with curcuminoids and tuna oil influenced skin yellowness, carcass composition, oxidation status, and meat fatty acids of slow-growing chickens. Poult. Sci..

[bib0024] Herd R., Arthur P. (2009). Physiological basis for residual feed intake. J. Anim. Sci..

[bib0025] Horodyska J., Wimmers K., Reyer H., Trakooljul N., Mullen A.M., Lawlor P.G., Hamill R.M. (2018). RNA-seq of muscle from pigs divergent in feed efficiency and product quality identifies differences in immune response, growth, and macronutrient and connective tissue metabolism. *BMC G**enom.*.

[bib0026] Jeong S.-J., Kim S., Park J.-G., Jung I.-h., Lee M.-N., Jeon S., Kweon H.Y., Yu D.-Y., Lee S.-H., Jang Y. (2018). Prdx1 (peroxiredoxin 1) deficiency reduces cholesterol efflux via impaired macrophage lipophagic flux. Autophagy.

[bib0027] Kadhim K.K., Zuki A., Noordin M., Babjee S., Khamas W. (2010). Growth evaluation of selected digestive organs from day one to four months post-hatch in two breeds of chicken known to differ greatly in growth rate. J. Anim. Vet. Adv..

[bib0028] Kohl J.B., Mellis A.T., Schwarz G. (2019). Homeostatic impact of sulfite and hydrogen sulfide on cysteine catabolism. Br. J. Pharmacol..

[bib0029] Kong B.-W., Lassiter K., Piekarski-Welsher A., Dridi S., Reverter-Gomez A., Hudson N.J., Bottje W.G. (2016). Proteomics of breast muscle tissue associated with the phenotypic expression of feed efficiency within a pedigree male broiler line: I. Highlight on mitochondria. PLoS One.

[bib0030] Kong R.S., Liang G., Chen Y., Stothard P. (2016). Transcriptome profiling of the rumen epithelium of beef cattle differing in residual feed intake. BMC genomics.

[bib0031] Krás R.V., Kessler A.d.M., Ribeiro A.M.L., Henn J.D., Bockor L., Sbrissia A.F. (2013). Effect of dietary fiber, genetic strain and age on the digestive metabolism of broiler chickens. Bra. J. Poult. Sci..

[bib0032] Lee J., Karnuah A.B., Rekaya R., Anthony N.B., Aggrey S.E. (2015). Transcriptomic analysis to elucidate the molecular mechanisms that underlie feed efficiency in meat-type chickens. Mol. Genet. Genom..

[bib0033] Lusk J.L. (2018). Consumer preferences for and beliefs about slow growth chicken. Poult. Sci..

[bib0034] Mabelebele M., Ng'ambi J., Norris D., Ginindza M. (2014). Comparison of gastrointestinal tract and pH values of digestive organs of Ross 308 broiler and indigenous Venda chickens fed the same diet. Asian J. Anim. Vet. Adv..

[bib0035] Mebratie W., Reyer H., Wimmers K., Bovenhuis H., Jensen J. (2019). Genome wide association study of body weight and feed efficiency traits in a commercial broiler chicken population, a re-visitation. Sci. Rep..

[bib0036] Mishra B., Jha R. (2019). Oxidative stress in the poultry gut: potential challenges and interventions. Front. Vet. Sci..

[bib0037] Nain S., Renema R., Zuidhof M., Korver D. (2012). Effect of metabolic efficiency and intestinal morphology on variability in n-3 polyunsaturated fatty acid enrichment of eggs. Poult. Sci..

[bib0038] N'Dri A.L., Mignon-Grasteau S., Sellier N., Tixier-Boichard M., Beaumont C. (2006). Genetic relationships between feed conversion ratio, growth curve and body composition in slow-growing chickens. Br. Poult. Sci..

[bib0039] Ojano-Dirain C., Iqbal M., Cawthon D., Swonger S., Wing T., Cooper M., Bottje W. (2004). Determination of mitochondrial function and site-specific defects in electron transport in duodenal mitochondria in broilers with low and high feed efficiency. Poult. Sci..

[bib0040] R Core Team (2020). https://www.R-project.org/.

[bib0041] Recoules E., Lessire M., Labas V., Duclos M.J., Combes-Soia L., Lardic L., Peyronnet C., Quinsac A., Narcy A., Réhault-Godbert S. (2019). Digestion dynamics in broilers fed rapeseed meal. Sci. Rep..

[bib0042] Richards M., Proszkowiec-Weglarz M. (2007). Mechanisms regulating feed intake, energy expenditure, and body weight in poultry. Poult. Sci..

[bib0043] Rougiere N., Gomez J., Mignon-Grasteau S., Carré B. (2009). Effects of diet particle size on digestive parameters in D+ and D− genetic chicken lines selected for divergent digestion efficiency. Poult. Sci..

[bib0044] Schäff C., Börner S., Hacke S., Kautzsch U., Albrecht D., Hammon H.M., Röntgen M., Kuhla B.r. (2012). Increased anaplerosis, TCA cycling, and oxidative phosphorylation in the liver of dairy cows with intensive body fat mobilization during early lactation. J. Proteome Res..

[bib0045] Shah T.M., Patel J.G., Gohil T.P., Blake D.P., Joshi C.G. (2019). Host transcriptome and microbiome interaction modulates physiology of full-sibs broilers with divergent feed conversion ratio. *NPJ Biofilms Microbiomes*.

[bib0046] Szklarczyk D., Franceschini A., Wyder S., Forslund K., Heller D., Huerta-Cepas J., Simonovic M., Roth A., Santos A., Tsafou K.P. (2015). STRING v10: protein–protein interaction networks, integrated over the tree of life. *Nucleic Acids Res**.*.

[bib0047] Tang Y., Horikoshi M., Li W. (2016). ggfortify: unified interface to visualize statistical results of popular R packages. R J..

[bib0048] Tyanova S., Temu T., Sinitcyn P., Carlson A., Hein M.Y., Geiger T., Mann M., Cox J. (2016). The Perseus computational platform for comprehensive analysis of (prote) omics data. Nat. Methods.

[bib0049] Wickham H. (2009). Elegant graphics for data analysis. Media.

[bib0050] Wu J., Wang X., Ding R., Quan J., Ye Y., Gu T., Xu Z., Zheng E., Cai G., Wu Z. (2020). Identification of important proteins and pathways affecting feed efficiency in DLY pigs by iTRAQ-based proteomic analysis. Animals.

[bib0051] Xiao C., Deng J., Zeng L., Sun T., Yang Z., Yang X. (2021). Transcriptome analysis identifies candidate genes and signaling pathways associated with feed efficiency in Xiayan chicken. Front. Genet..

[bib58] Yang C., Han L., Li P., Ding Y., Zhu Y., Huang Z., Dan X., Shi Y., Kang X. (2021). Characterization and duodenal transcriptome analysis of chinese beef cattle with divergent feed efficiency using RNA-seq. Front. Genet..

[bib0053] Yang L., He T., Xiong F., Chen X., Fan X., Jin S., Geng Z. (2020). Identification of key genes and pathways associated with feed efficiency of native chickens based on transcriptome data via bioinformatics analysis. *BMC Genom**.*.

[bib0054] Yi G., Yuan J., Bi H., Yan W., Yang N., Qu L. (2015). In-depth duodenal transcriptome survey in chickens with divergent feed efficiency using RNA-Seq. PloS One.

[bib0055] Zhang J., Shi H., Li S., Cao Z., Yang H., Wang Y. (2019). Integrative hepatic metabolomics and proteomics reveal insights into the mechanism of different feed efficiency with high or low dietary forage levels in Holstein heifers. J. Proteom..

[bib0057] Zhao D., Kogut M.H., Genovese K.J., Hsu C.-Y., Lee J.T., Farnell Y.Z. (2020). Altered expression of lactate dehydrogenase and monocarboxylate transporter involved in lactate metabolism in broiler wooden breast. Poult. Sci..

