# Supplementary figures and images for: Comparative proteomics revealed duodenal metabolic function associated with feed efficiency in slow-growing chicken
Source: Poult Sci. 2022 Mar 8;101(6):101824. doi: 10.1016/j.psj.2022.101824 (PMC8987610; doi:10.1016/j.psj.2022.101824)

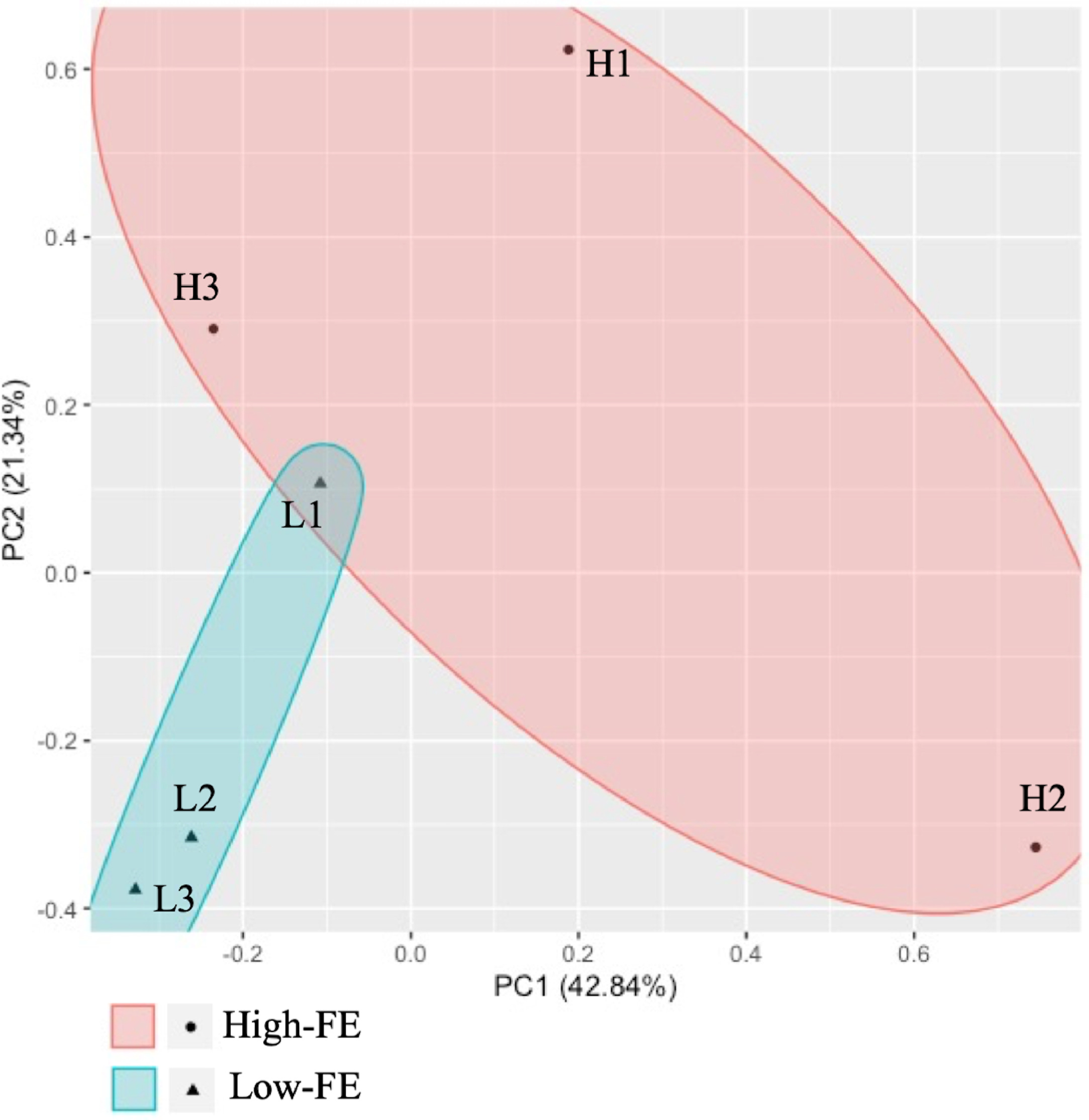

Supplement: Supplementary file 2 — Supplementary Figure 1. Principal component analysis (PCA) plot using 355 proteins from the low-FE (blue) and high-FE (red) groups. [file mmc2.jpg]

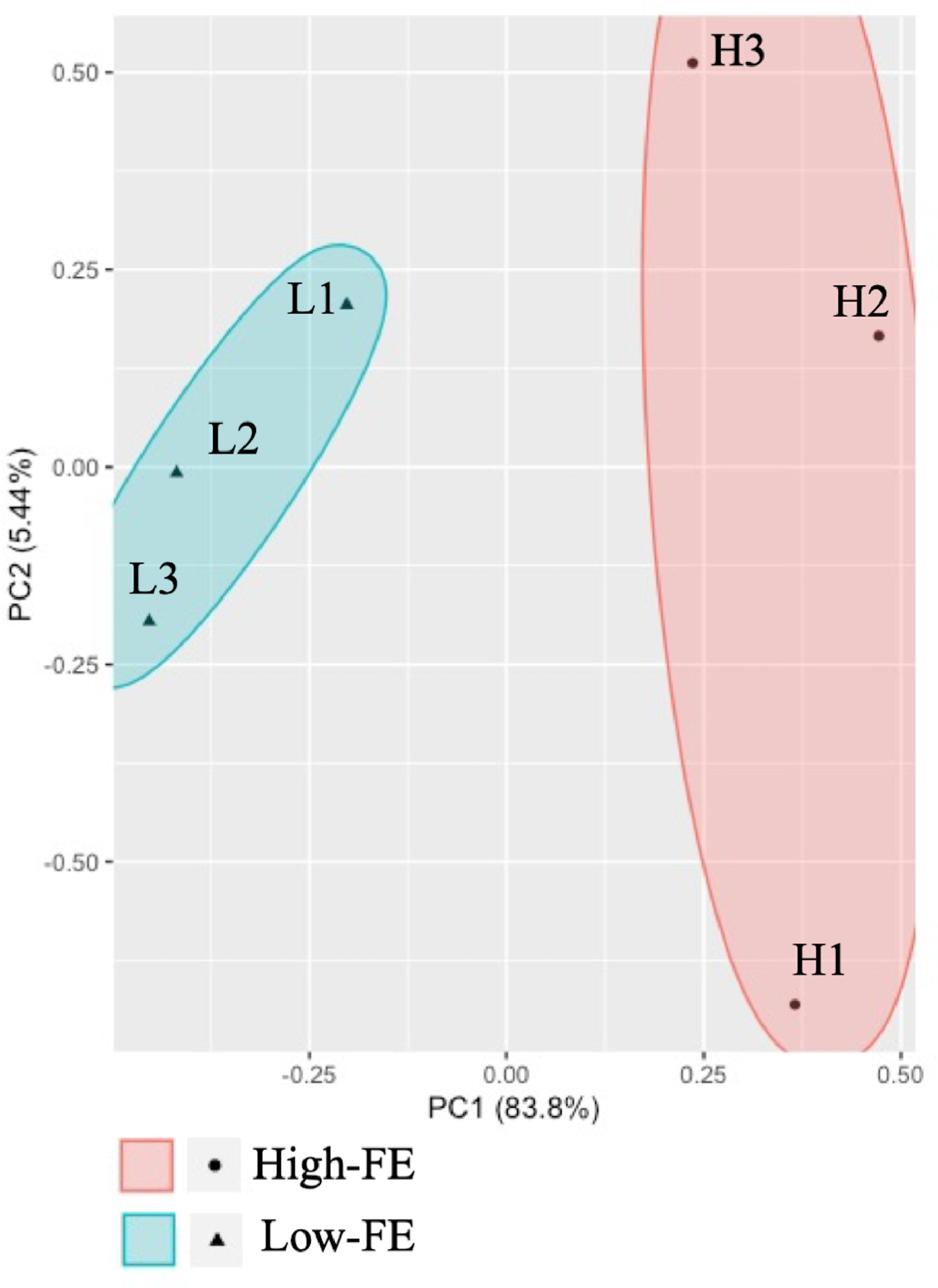

Supplement: Supplementary file 3 — Supplementary Figure 2. Principal component analysis (PCA) plot using 40 DAPs from the low-FE (blue) and high-FE (red) groups. [file mmc3.jpg]
